# Recent Advances in Understanding FOXN3 in Breast Cancer, and Other Malignancies

**DOI:** 10.3389/fonc.2019.00234

**Published:** 2019-05-31

**Authors:** Xiangyi Kong, Jie Zhai, Chengrui Yan, Yan Song, Jing Wang, Xiaofeng Bai, James A. L. Brown, Yi Fang

**Affiliations:** ^1^Department of Breast Surgical Oncology, National Cancer Center/National Clinical Research Center for Cancer/Cancer Hospital, Chinese Academy of Medical Sciences and Peking Union Medical College, Beijing, China; ^2^Department of Neurosurgery, Peking University International Hospital, Beijing, China; ^3^Department of Pathology, National Cancer Center/National Clinical Research Center for Cancer/Cancer Hospital, Chinese Academy of Medical Sciences and Peking Union Medical College, Beijing, China; ^4^Department of Pancreatic-Gastric Surgery, National Cancer Center/National Clinical Research Center for Cancer/Cancer Hospital, Chinese Academy of Medical Sciences and Peking Union Medical College, Beijing, China; ^5^Discipline of Surgery, School of Medicine, Lambe Institute for Translational Research, National University of Ireland Galway, Galway, Ireland; ^6^Centre for Chromosome Biology, National University of Ireland in Galway, Galway, Ireland

**Keywords:** FOXN3, forkhead, function, breast, cancer, malignancy, regulation, review

## Abstract

FOXN3 (forkhead box N3; CHES1: check point suppressor 1) belongs to the forkhead box (FOX) protein family. FOXN3 displays transcriptional inhibitory activity, and is involved in cell cycle regulation and tumorigenesis. FOXN3 is a tumor suppresser and alterations in FOXN3 are found in of a variety of cancers including melanoma, osteosarcoma, and hepatocellular carcinoma. While the roles of FOXN3 role in some cancers have been explored, its role in breast cancer remains unclear. Here we describe current state of knowledge of FOXN3 functions, and focus on its roles (known and potential) in breast cancer.

## Introduction

The Forkhead proteins (FOX) belongs to a subgroup of “helical-fold-helical” proteins (1). At the molecular level FOX proteins act as monomers directing transcription (through DNA binding), acting as a platform to coordinate transcription factors (2). Through this activity FOX proteins influence nucleosome location and structure, promoting gene activation (3). The DNA binding domain of the FOX proteins (“forkhead box”) is a highly conserved 100 amino acids region composed of three α helices (H1, H2, and H3) and two characteristic loop structures. These form a characteristic wing-like helix structure (“wing-shaped helix”), where the H3 motif represents the main recognition and DNA binding site (4, 5). Based on homology of DNA binding domains, it has been determined that the FOX protein has over 100 family members in different species of eukaryotic cells, including 17 subfamilies (families A-Q) (Tables 1, 2) (6–8).

**Table 1:** Forty-eight FOX family proteins are classified into 19 subfamilies.

**Subfamily**	**Subfamily members**
FOXA	FOXA1, FOXA, FOXA
FOXB	FOXB1, FOXB2
FOXC	FOXC1, FOXC2
FOXD	FOXD1, FOXD2, FOXD3, FOXD4, FOXD4L1, FOXD4L3
FOXE	FOXE1, FOXE3
FOXF	FOXF1, FOXF2
FOXG	FOXG1
FOXH	FOXH1
FOXI	FOXI1, FOXI2, FOXI3
FOXJ	FOXJ1, FOXJ2, FOXJ3
FOXK	FOXK1, FOXK2
FOXL	FOXL1, FOXL2
FOXM	FOXM1
FOXN	FOXN1, FOXN2, FOXN3, FOXN4, FOXN5, FOXN6
FOXO	FOXO1, FOXO2, FOXO3, FOXO4
FOXP	FOXP1, FOXP2, FOXP3, FOXP4
FOXQ	FOXQ1
FOXR	FOXR1, FOXR2
FOXS	FOXS1

**Table 2:** FOX family members' gene card information.

**Gene**	**Genomic location**	**Important paralog**	**Associated disorders**	**Related pathways**	**Gene Ontology (GO) annotations**	**Top transcription factor binding sites**
FOXA1	14q21.1	FOXA2	EP Positive Breast Cancer and Luminal Breast Carcinoma	Direct p53 effectors and FOXA1 transcription factor network	DNA binding transcription factor activity and transcription factor binding	FOXO4, HTF, PPAR-alpha, Sp1, ATF, FOXD3, GATA-1, NRSF, form, 1, NRSF, form, 2, Sox9
FOXA2	20p11.21	FOXA1	Meckel Diverticulum and Maturity-Onset Diabetes of The Young	Regulation of beta-cell development and Longevity regulating pathway	DNA binding transcription factor activity and transcription factor binding	HFH-1, AP-2alphaA, AP-2alpha, S8, ATF
FOXA3	19q13.32	FOXA1	No Data Available	Regulation of beta-cell development and FOXA1 transcription factor network	DNA binding transcription factor activity and transcription factor binding	Olf-1, USF1, USF-1, Ik-3, ZID, E2F-1, E2F, NF-1, NF-1/L
FOXB1	15q22.2	FOXB2	No Data Available	No Data Available	DNA binding transcription factor activity, RNA polymerase II transcription factor activity, sequence-specific DNA binding	E2F-1 E2F Max E2F-2 E2F-3a E2F-4 E2F-5 S8 HFH-1 POU2F1
FOXB2	9q21.2	FOXB1	No Data Available	No Data Available	DNA binding transcription factor activity, RNA polymerase II transcription factor activity, sequence-specific DNA binding	Nkx6-1 FOXL1 HTF Hlf Sox5 POU2F1c POU2F1 POU2F1a POU2F1b Chx10
FOXC1	6p25.3	FOXC2	Anterior Segment Dysgenesis 3 and Axenfeld-Rieger Syndrome, Type 3	Transcriptional Regulatory Network in Embryonic Stem Cell and Heart Development	DNA binding transcription factor activity and transcription factor binding	STAT1, FOXO1a, FOXO1, FOXO4, SRF, (504, AA), SRF, Nkx2-5
FOXC2	16q24.1	FOXC1	Lymphedema-Distichiasis Syndrome and Distichiasis	Adipogenesis and Glucose/Energy Metabolism	DNA binding transcription factor activity and transcription regulatory region DNA binding	MyoD, C/EBPalpha, Pax-4a
FOXD1	5q13.2	FOXD2	Hemophagocytic Lymphohistiocytosis, Familial, 2	Preimplantation Embryo	DNA binding transcription factor activity and RNA polymerase II proximal promoter sequence-specific DNA binding	FOXO4, FOXO1a, FOXO1, ATF, Nkx2-5, Egr-3, FOXJ2, (long, isoform), FOXJ2
FOXD2	1p33	FOXD1	No Data Available	No Data Available	DNA binding transcription factor activity and RNA polymerase II transcription factor activity, sequence-specific DNA binding	Sox9, Egr-4, Nkx2-5, RP58, Egr-1, RREB-1, C/EBPalpha, AP-4, GATA-2, CREB
FOXD3	1p31.3	FOXD1	Autoimmune Disease 1 and Senile Entropion	Transcriptional Regulatory Network in Embryonic Stem Cell and Oct4 in Mammalian ESC Pluripotency	DNA binding transcription factor activity and transcription regulatory region DNA binding	E2F, LHX3b, E2F-1, E2F-2, E2F-3a, E2F-4, E2F-5, CUTL1, LCR-F1, Lhx3a
FOXD4	9p24.3	FOXD4L3	Obsessive-Compulsive Disorder and Dilated Cardiomyopathy	No Data Available	DNA binding transcription factor activity and RNA polymerase II transcription factor activity, sequence-specific DNA binding	FOXO4, LCR-F1, GATA-2, TBP, C/EBPbeta, Pax-3, STAT3, GATA-1
FOXD4L1	2q14.1	FOXD4	No Data Available	No Data Available	DNA binding transcription factor activity and RNA polymerase II transcription factor activity, sequence-specific DNA binding	FOXO4, LCR-F1, GATA-2, p53, TBP, C/EBPbeta, Pax-3, STAT3, GATA-1, CP2
FOXD4L3	9q21.11	FOXD4L6	No Data Available	No Data Available	DNA binding transcription factor activity and RNA polymerase II transcription factor activity, sequence-specific DNA binding	RREB-1, GATA-2, MyoD, TBP, FOXD1, C/EBPbeta, p53, STAT3, GATA-1, aMEF-2
FOXE1	9q22.33	FOXE3	Hypothyroidism, Thyroidal Or Athyroidal, With Spiky Hair And Cleft Palate and Thyroid Cancer, Non-medullary, 4	No Data Available	DNA binding transcription factor activity and RNA polymerase II transcription factor activity, sequence-specific DNA binding	FOXO4, FOXO1a, FOXO1, Pax-4a, NRSF, form, 2, NRSF, form, 1, Nkx6-1, p53, GR, GR-alpha
FOXE3	1p33	FOXE1	Anterior Segment Dysgenesis 2 and Cataract 34, Multiple Types	No Data Available	DNA binding transcription factor activity and RNA polymerase II transcription factor activity, sequence-specific DNA binding	FOXO4, RP58, Nkx2-5, CP2, deltaCREB, CREB, c-Ets-1, Ik-1, HOXA3, GR
FOXF1	16q24.1	FOXF2	Alveolar Capillary Dysplasia with Misalignment Of Pulmonary Veins and Persistent Fetal Circulation Syndrome	Embryonic and Induced Pluripotent Stem Cell Differentiation Pathways and Lineage-specific Markers and FOXA2 and FOXA3 transcription factor networks	DNA binding transcription factor activity and transcription regulatory region DNA binding	p53, FOXO4, FOXO1a, FOXO1, MyoD, STAT3, E2F
FOXF2	6p25.3	FOXF1	Epicanthus	No Data Available	DNA binding transcription factor activity and transcription factor binding	FOXO1, FOXO1a, CHOP-10, E47, Hand1, C/EBPalpha, Pax-3, GATA-1
FOXG1	14q12	FOXD2	Rett Syndrome, Congenital Variant and Rett Syndrome	FoxO signaling pathway and Regulation of nuclear SMAD2/3 signaling	DNA binding transcription factor activity and sequence-specific DNA binding	FOXO4, POU2F1, HFH-1, Oct-B1, oct-B2, oct-B3, POU2F1a, POU2F2, POU2F2, (Oct-2.1)
FOXH1	8q24.3	FOXF2	Microform Holoprosencephaly and Lobar Holoprosencephaly	SMAD Signaling Network and Signaling by NODAL	DNA binding transcription factor activity and protein domain specific binding	p53, Sp1, Nkx2-5, AP-2alpha
FOXI1	5q35.1	FOXI3	Deafness, Autosomal Recessive 4, With Enlarged Vestibular Aqueduct and Pendred Syndrome	No Data Available	DNA binding transcription factor activity and RNA polymerase II proximal promoter sequence-specific DNA binding	deltaCREB, CREB, Sp1, Zic3, PPAR-gamma2, PPAR-gamma1, NRSF, form, 2
FOXI2	10q26.2	FOXI1	Noonan Syndrome 1	No Data Available	DNA binding transcription factor activity and RNA polymerase II transcription factor activity, sequence-specific DNA binding	NF-kappaB, NF-kappaB1, STAT1alpha, STAT1, STAT1beta, STAT2, STAT3, STAT4, STAT5A, STAT5B
FOXI3	2p11.2	FOXI1	No Data Available	No Data Available	DNA binding transcription factor activity and sequence-specific DNA binding	Evi-1, PPAR-gamma2, PPAR-gamma1, Elk-1, COUP-TF, COUP, COUP-TF1, HNF-4alpha1, HNF-4alpha2, HOXA9B
FOXJ1	17q25.1	FOXJ3	Rhinitis and Allergic Rhinitis	Embryonic and Induced Pluripotent Stem Cell Differentiation Pathways and Lineage-specific Markers	DNA binding transcription factor activity and RNA polymerase II transcription factor activity, sequence-specific DNA binding	FOXO1a, FOXO1, FOXO4, RREB-1, Nkx5-1, NF-kappaB1
FOXJ2	12p13.31	FOXJ3	Cardiomyopathy, Familial Hypertrophic, 3	No Data Available	DNA binding transcription factor activity and RNA polymerase II proximal promoter sequence-specific DNA binding	FOXO4, Elk-1, YY1, NF-kappaB, NF-kappaB1, HOXA9, HOXA9B, Meis-1, Meis-1b, Nkx6-1
FOXJ3	1p34.2	FOXJ2	No Data Available	No Data Available	DNA binding transcription factor activity and RNA polymerase II transcription factor activity, sequence-specific DNA binding	FOXO1, FOXO1a, FOXO4, COUP-TF1, COUP-TF, COUP, HNF-4alpha1
FOXK1	7p22.1	FOXK2	Thyroid Angiosarcoma and Thyroid Sarcoma	Metabolism of proteins and Deubiquitination	DNA binding transcription factor activity and RNA polymerase II transcription factor activity, sequence-specific DNA binding	FOXO1a, FOXO1, C/EBPalpha, Ik-3, NF-E2, p45, NF-E2, RelA, NF-kappaB1, NF-kappaB, ATF
FOXK2	17q25.3	FOXK1	No Data Available	Metabolism of proteins and Wnt/Hedgehog/Notch	DNA binding transcription factor activity and RNA polymerase II proximal promoter sequence-specific DNA binding	c-Ets-1, AML1a, STAT5A, MRF-2, Pax-5, PPAR-alpha, E2F-1, E2F, CUTL1, CREB
FOXL1	16q24.1	FOXC1	Lymphedema-Distichiasis Syndrome and Hypoplastic Left Heart Syndrome	Ectoderm Differentiation	DNA binding transcription factor activity and RNA polymerase II transcription factor activity, sequence-specific DNA binding	MyoD, C/EBPalpha, ZID, Pax-4a, PPAR-gamma2
FOXL2	3q22.3	FOXC1	Blepharophimosis, Ptosis, And Epicanthus Inversus and Premature Ovarian Failure 3	No Data Available	DNA binding transcription factor activity and RNA polymerase II transcription factor activity, sequence-specific DNA binding	FOXO4, FOXO1a, FOXO1, E2F-1, E2F, E2F-2, E2F-3a
FOXM1	12p13.33	FOXO3	Hepatocellular Carcinoma and Meckel Diverticulum	Cell Cycle, Mitotic and Sudden Infant Death Syndrome (SIDS) Susceptibility Pathways	DNA binding transcription factor activity and protein kinase binding	deltaCREB, CREB, YY1, C/EBPalpha
FOXN1	17q11.2	FOXN4	T-Cell Immunodeficiency, Congenital Alopecia, And Nail Dystrophy and Alopecia	No Data Available	DNA binding transcription factor activity and RNA polymerase II transcription factor activity, sequence-specific DNA binding	NF-kappaB, NF-kappaB1, SRF, Olf-1, Nkx2-5, SRF, (504, AA), c-Myc, Max1, AREB6
FOXN2	2p16.3	FOXN3	T-Cell Leukemia	No Data Available	DNA binding transcription factor activity and sequence-specific DNA binding	Lmo2, AP-4 E47, FOXO1a, FOXO1, FOXO4
FOXN3	14q31.3-q32.11	FOXN2	No Data Available	Mesodermal Commitment Pathway	DNA binding transcription factor activity and protein C-terminus binding	FOXO4, AP-2alphaA, AP-2alpha, isoform, 4, AP-2alpha, isoform, 3, AP-2alpha, isoform, 2, AP-2alpha, Bach2, AREB6, C/EBPalpha
FOXN4	12q24.11	FOXN1	No Data Available	No Data Available	DNA binding transcription factor activity and chromatin binding	AML1a, Ik-3, FOXO1, FOXO1a, FOXO4
FOXO1	13q14.11	FOXO3	Rhabdomyosarcoma 2 and Glioma	Akt Signaling and Integrated Breast Cancer Pathway	DNA binding transcription factor activity and chromatin binding	AP-1, COUP, COUP-TF, COUP-TF1, HNF-4alpha1
FOXO3	6q21	FOXO1	Chromosome 6Q Deletion and Rhabdomyosarcoma	HIV Life Cycle and TRAF Pathway	DNA binding transcription factor activity and protein kinase binding	STAT5A, AP-1
FOXO4	Xq13.1	FOXO1	Balloon Cell Malignant Melanoma and Sarcomatoid Squamous Cell Skin Carcinoma	EGF/EGFR Signaling Pathway and HIV Life Cycle	DNA binding transcription factor activity and enzyme binding	GR-alpha, GR, Evi-1, GR-beta, Tal-1, c-Myb, E47, IRF-2, HFH-1
FOXP1	3p13	FOXP2	Mental Retardation with Language Impairment and With or Without Autistic Features and Lymphoma, Mucosa-Associated Lymphoid Type	MicroRNAs in cancer and Wnt / Hedgehog / Notch	DNA binding transcription factor activity and sequence-specific DNA binding	NF-kappaB1, NF-kappaB, aMEF-2, RelA, COMP1, MEF-2A, Nkx3-1, Nkx3-1, v1
FOXP2	7q31.1	FOXP1	Childhood Apraxia of Speech and Speech and Communication Disorders	Wnt / Hedgehog / Notch and Pathways Affected in Adenoid Cystic Carcinoma	DNA binding transcription factor activity and sequence-specific DNA binding	FOXO1, FOXO1a
FOXP3	Xp11.23	FOXP4	Immunodysregulation, Polyendocrinopathy, And Enteropathy, X-Linked and Diabetes Mellitus, Insulin-Dependent	Th2 Differentiation Pathway and Wnt / Hedgehog / Notch	DNA binding transcription factor activity and sequence-specific DNA binding	CREB, deltaCREB, AML1a
FOXP4	6p21.1	FOXP2	No Data Available	No Data Available	DNA binding transcription factor activity and sequence-specific DNA binding	Pax-3, Sox9, FOXO3b, FOXO3a, FOXO3, Pax-5, Pbx1a, CUTL1, FOXD1, NRSF
FOXQ1	6p25.3	FOXD2	Ritscher-Schinzel Syndrome	Preimplantation Embryo	DNA binding transcription factor activity and RNA polymerase II transcription factor activity, sequence-specific DNA binding	AML1a, LCR-F1, RFX1, Pax-5
FOXR1	11q23.3	FOXR2	No Data Available	No Data Available	DNA binding transcription factor activity and RNA polymerase II transcription factor activity, sequence-specific DNA binding	p53, RP58, MAZR, Roaz, Pax-4a, S8, AML1a, SEF-1 (1), Spz1, deltaCREB
FOXR2	Xp11.21	FOXR1	No Data Available	No Data Available	DNA binding transcription factor activity and RNA polymerase II transcription factor activity, sequence-specific DNA binding	FOXO1a, FOXO1, FOXO4, GR, GR-alpha, GR-beta, AML1a, STAT1alpha, STAT1beta, STAT2
FOXS1	20q11.21	FOXC2	No Data Available	No Data Available	DNA binding transcription factor activity and RNA polymerase II transcription factor activity, sequence-specific DNA binding	Nkx2-5, GATA-2, AhR, AML1a, SRF, SRF, (504, AA), GCNF-2, GCNF-1, GCNF, NF-kappaB

As transcriptional regulators, the FOX protein family regulates the development of tissues, embryos as well as carbohydrate and lipid metabolism (9–11). Furthermore, FOX proteins are involved in regulating biological processes including the cell cycle (5, 12), homeostasis and immunization (13). Dysregulation of FOX family members can cause developmental malformation of tissues and organs, metabolic diseases, and importantly is associated with the development of tumors (14, 15). Down-regulation of the FOXO subfamily (regulating signal transduction pathways) is found in multiple tumors (including breast, prostate, glioblastoma, hematological malignancy, alveolar Rhabdomyosarcoma), leading to aberrant apoptosis, cell cycle, and DNA repair defects (16, 17). As many FOX proteins directly interact with kinases in multiple signaling pathways, they are attractive therapeutic targets for many diseases. Here we focus on the FOXN subfamily due to that the FOXN genes remain an understudied forkhead subfamily with little known about the transcriptional activity of the proteins within this group.

**Figure 1:**
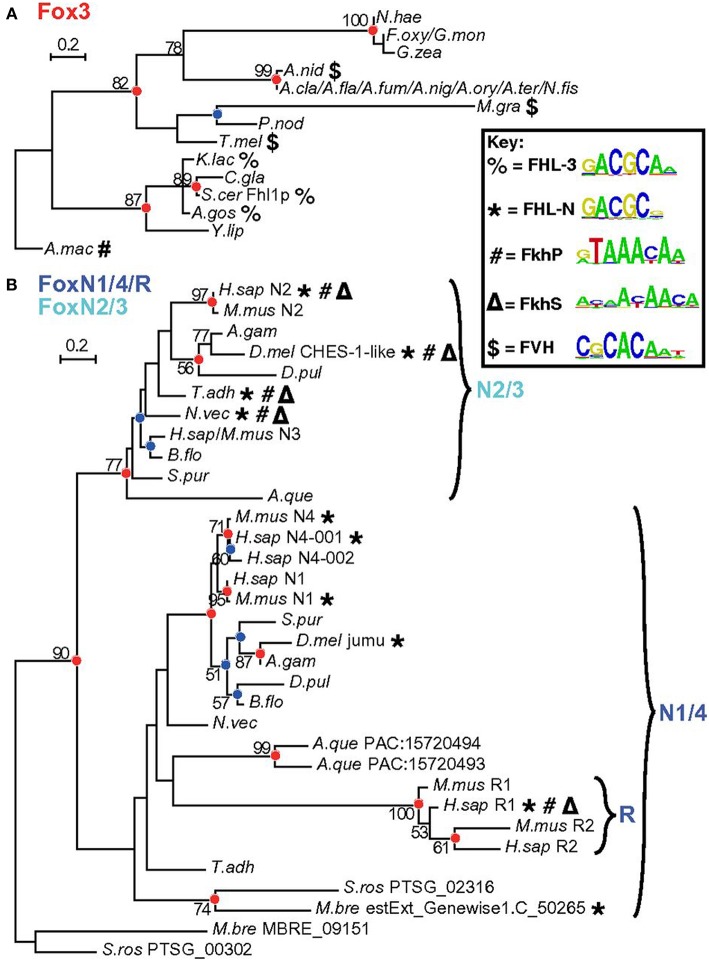
Detailed analysis of Fox3 and FoxN subfamilies. Maximum likelihood (ML) phylogenetic trees for Fox domains from a broader range of species for fungal Fox3 **(A)** and holozoan FoxN/R **(B)** clades. Red and blue circles indicate corresponding nodes. Bold symbols represent binding capacity for different motif classes. Reprinted from Nakagawa et al. (18). Copyright® 2013 National Academy of Sciences.

FOXN3 is among the few FOX family members that binds both a FORKHEAD (FKH) and a FORKHEAD-LIKE (FHL) sequence. A nice series of dendograms has been presented by Nakagawa et al. (18) (Figure 1). Rogers et al. has very recently (27 February, 2019) solved the crystal structures of FOXN3 bound to both FKH and FHL sites, respectively, and made very interesting observations: the same protein domain binds two different DNA sequences because the FOX domain recognizes the totality (sequence and backbone) of the element, and can bend it substantially (19). However, the DNA structure is different in the two complexes. These structures reveal how a single transcription factor binds two unrelated DNA sequences and the importance of DNA shape in the mechanism of bispecific recognition. Also, from the discovery, we could understand how the vast majority (around 80%) of precipitated chromatin in the ChIP-Seq experiment did not have either a FKH (around 18%) or a FHL (around 2–3%) sequence: FOXN3 mostly binds DNA through interaction with a partner (19).

## FOXN Family

### FOXN Family Overview

**Figure 2:**
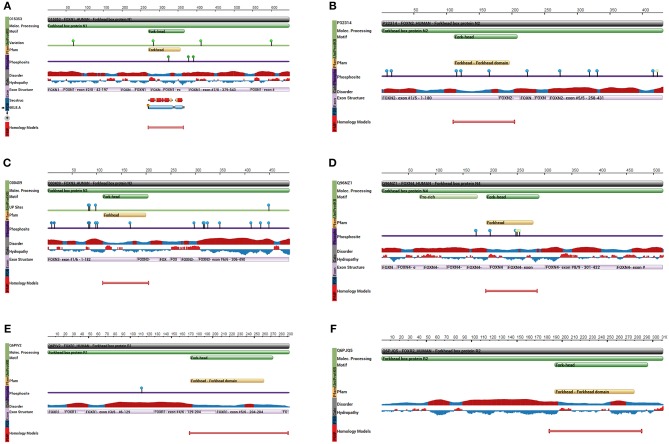
Key protein features for the indicated FOXN proteins. **(A)** The protein feature view for the FOXN1 protein. **(B)** The protein feature view for FOXN2 protein. **(C)** The protein feature view for FOXN3 protein. **(D)** The protein feature view for FOXN4 protein. **(E)** The protein feature view for FOXN5 protein. **(F)** The protein feature view for FOXN6 protein. All protein structures generated using the Protein Data Bank (RCSB PDB; http://www.rcsb.org/pdb/protein/).

As a subfamily of the FOX family transcription factors, the FOXN protein family consists of six members: FOXN1 (Figure 2A), FOXN2 (HTLF) (Figure 2B), FOXN3 (CHES1) (Figure 2C), FOXN4 (Figure 2D), FOXN5 (FOXR1) (Figure 2E), and FOXN6 (FOXR2) (Figure 2F) (20). FOXN1 (WHN) regulates the growth of the keratinized epithelial cells and the differentiation of thymic epithelial cells required for T cell development (21, 22). In addition, FOXN1 is regulated by Wnt glycoproteins, important for regulating keratinocyte growth and differentiation of thymic epithelium (23).

FOXN2 (human T-cell leukemia factor: HTLF) was identified as binding to the long-chain terminal repeat sequence of the human T-cell leukemia virus (HTLV-ILTR), participating in HTLV-ILT transcriptional regulation (24).

FOXN3 is the primary focus of this review, and will be discussed extensively below in the following sections.

FOXN4 is involved in the formation of amacrine cells and horizontal cells in the retina, and can upregulate the expression of specific retinal factors (25). The chimeric expression of FOXN4 with its mutants may serve as a trigger, thereby ensuring the diversity of the neurons in the V2 domain of the ventral spinal cord. For instance, FOXN4^lacZ/lacZ^ is an important mutant type of FOXN4. Li etc. showed that during murine lung development the FOXn4 transcription factor is expressed in proximal airways by a subpopulation of postmitotic epithelial cells which are distinct from basal and ciliated cells and of which only a small fraction are Clara cells. FOXN4^lacZ/lacZ^ causes dilated alveoli, thinned alveolar walls and reduced septa in the distal lung but no overt gross alterations in proximal airways. The alveolar defects in mutants may result from decreased PDGFA signaling and reduced surfactant SFTPB expression (26).

Additionally, FOXN4 can regulate differentiation of the multi-center cells (MCC), and is involved in the development of multiple organelles including nucleus and mitochondria (25).

FOXN5 (FOXR1) was originally reported as a candidate tumor suppressor gene, with mutations leading to abnormal structural changes closely associated with the occurrence of neuroblastoma (27). FOXN5 is a Protein Coding gene. Gene Ontology (GO) annotations related to this gene include DNA binding transcription factor activity and RNA polymerase II transcription factor activity, sequence-specific DNA binding.

FOXN6 (FOXR2) regulates the proliferation of the hepatocellular carcinoma cells, and dysregulation promotes tumor formation in chronic hepatocellular carcinoma (HCC). It is believed that FOXN6 is a new promising therapeutic target for HCC (28). They reported that FOXN6 is frequently up-regulated in 25/42 (59.5%) of HCC specimens compared with neighboring non-cancerous tissues in messenger RNA (mRNA) level and further confirmed by immunohistochemistry analysis in protein level. Moreover, FOXN6 overexpression facilitated the development of tumor xenografts in nude mice model (28).

### FOXN3 Overview

FOXN3 (CHES1, PRO1635, or C14orf116) was originally isolated by Pati et al. in yeast cells with multiple checkpoint mutations (29). FOXN3 suppresses a number of DNA damage-activated checkpoint mutations in *S. cerevisiae*, including mec1, rad9, rad24, dun1, and rad53. FOXN3 suppression of sensitivity to DNA damage is specific for checkpoint-defective strains, in contrast to DNA repair-defective strains. Presence of FOXN3 but not a control vector resulted in G2 delay after UV irradiation in checkpoint-defective strains, with kinetics, nuclear morphology, and cycloheximide resistance similar to those of a wild-type strain. FOXN3 can also suppress the lethality, UV sensitivity, and G2 checkpoint defect of a mec1 null mutation. In contrast to this activity, FOXN3 had no measurable effect on the replication checkpoint as assayed by hydroxyurea sensitivity of a mec1 strain (29). The FOXN3 gene (in humans located at 14q31.3 ~ q32. 11), is 463bp long and contains 7 exons. The protein symbol for FOXN3 gene is O00409-FOXN3_HUMAN with a size of 490 amino acids, which interacts through its C-terminus with the C-terminus of SNW1/SKIP. The structure of FOXN3 is shown in Figure 2C. FOXN3 is the only member of the FOXN family with no transcriptional binding domain (lacking transcriptional activation domains), while its carboxyl terminal serves as the transcriptional repressor (30, 31). Fusing the carboxyl terminal of FOXN3 onto a heterologous DNA binding domain allows continuous suppression of target genes (32).

In humans FOXN3 is widely expressed in many organs (including the liver, pancreas, kidneys, lungs and bone marrow), where FOXN3 plays indispensable roles in the development of these tissues (32, 33). This was found to be true in other vertebrates, where Schmidt et al. confirmed in animal models of *Xenopus laevis* and carp larvae that FOXN3 is critical for the development of new cartilage in the brain, and influences the development of muscle morphology indirectly (34). FOXN3 was found in studies on the regulation of fasting blood glucose, with patients carrying the SNP (rs8004664) found to have gene overexpression under fasting state. This up-regulation of FOXN3 leads to increased inhibition of the Myc gene (by FOXN3), and stimulates FOXN3 regulation of blood glucose levels. This role lead to FOXN3 being defined as a pathomechanical regulator of the fasting blood glucose (11). A paradigm-shifting study on FOXN3 intron polyadenylation in leukemia that appeared in August 2018 in Nature discussed FOXN3 intron polyadenylation, and confirms that MYC is a direct repressive target of FOXN3 (35). The tumor-suppressor gene (TSG) MGA is targeted by phenocopy truncating mutations in chronic lymphocytic leukemia (CLL) and solid cancers. MGA negatively regulates the MYC transcriptional program and represses genes with MYC- and E2F-binding sites in a Polycomb-dependent manner (35). Expression of MGA from constructs validated MGA intronic polyadenylation detected in CLL cells and confirmed the repressive effect of MGA on MYC target gene expression in malignant B cells (35). Notably, on genes with binding sites for both MYC and E2F, MGA IPA acts as dominant-negative regulator of full-length MGA as it significantly induced the expression of 5 out of 6 genes in cells that endogenously express fulllength MGA. In addition, the IPA isoform of the transcriptional repressor FOXN3 derepressed its oncogenic targets MYC and PIM2 (35).

**Figure 3:**
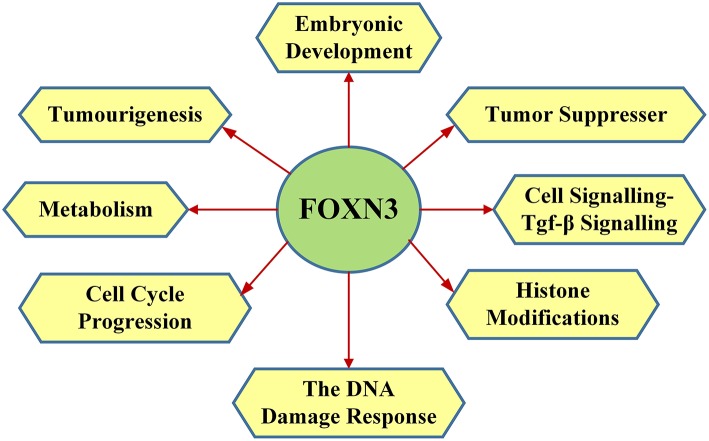
The main functions of FOXN3 including embryonic development, tumor suppresser, cell signalling-TGF-β signaling, histone modifications, the DNA damage response, cell cycle progression, metabolism, and tumourigenesis.

FOXN3 functions in the DNA damage response, where FOXN3 is responsible for S phase cell cycle arrest (in drosophila) (36–38) Upon DNA damage FOXN3 can induce quiescence, allowing damaged cells to persist (33). In yeast cells with cell cycle checkpoint defect (such as mutations of mec1, rad24, dun1, rad9, and rad53), FOXN3 can bind to the Sin protein in the Sin3/Rpd3 HDAC (histone deacetylation) complex to inhibit deacetylation of histones. This facilitates DNA damage repair and a G2/M phase arrest, thereby compensating for the cell cycle checkpoint defects (39). Checkpoints are eukaryotic DNA damage-inducible cell cycle arrests at G1 and G2. Checkpoint suppressor 1 suppresses multiple yeast checkpoint mutations including mec1, rad9, rad53, and dun1 by activating a MEC1-independent checkpoint pathway. Alternative splicing is observed at the locus, resulting in distinct isoforms. The main functions of FOXN3 are shown in Figure 3.

## FOXN3 and TGF-β Signaling

**Figure 4:**
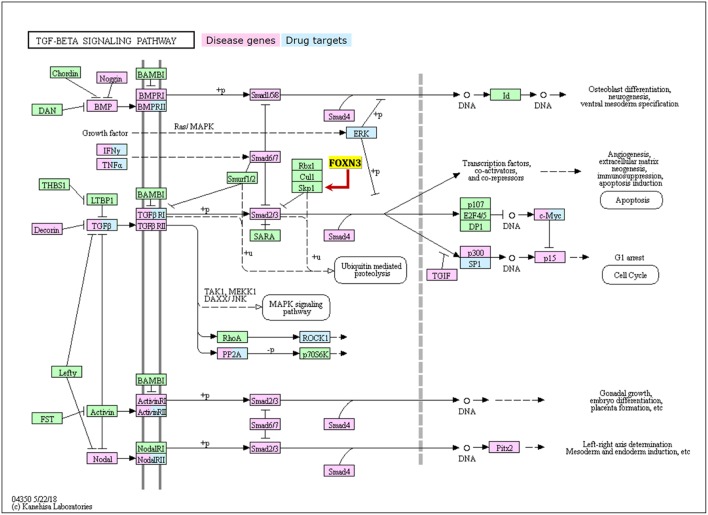
The TGF-β signalling pathway with showing key disease genes, drug targets, and the regulation relationship with FOXN3.

The transforming growth factor β (TGF-β) 1 superfamily members represent multifunctional cell signaling proteins that include TGF-β, bone morphogenetic protein (BMP), activin, inhibin, and growth differentiation factors. As such, the TGF-β1 family regulates many key cellular processes during growth and development (Figure 4). The TGF-β signaling pathway sequentially activates Smad 2 and Smad 3 by binding to two cell surface receptors (serine and threonine kinases), so that they can be translocated together with Smad4 to the nucleus, prompting the binding between Smad heterodimer and Smad binding element (SBE) and acting on the promoter of the target gene together with other nuclear factors (40). The Smad proteins also interact with other nuclear factors (such as the Ski, Sno, and nuclear hormone receptors), thereby regulating the transduction of TGF-β mediated signaling.

Mutations in Ski and Sno lead to oncogenic transformations, by blocking the transduction of the TGF-β signaling (41). The SKIP protein, a highly conserved transcription adaptor protein, is a nuclear hormone receptor co-activator. It was named initially for its recognition of the two-hybrid screening protein interacting with the V-Ski proto-oncogene. The protein can regulate the proliferation and differentiation of cells, and is involved in the transduction of multiple signaling pathways (42). A study in liver and melanoma malignancy cells (where FOXN3 expression has been reported to be decreased compared to corresponding normal tissue) showed that FOXN3 protein can act on the SKIP through its COOH terminal, repressing SKIP mediated TGF-β signaling (42).

In the TGF-β/smad cell signaling pathway, the Ski protein, as an inhibitor of the Smad2/3 protein transcription complex, inhibits the promoter of TGF-β by interacting with the Smad2/3 proteins, thereby inhibiting TGF-β/smad signal transduction and attenuating the anti-tumor effects of the TGF-β signaling pathway (43).

SKIP is an activator of Smad, in contrast to repressive Ski, competing with Ski to up-regulate the TGF-β signaling (44, 45). It has been proposed that this function of SKIP is a tumor-suppressive regulation of the TGF-β signaling pathway (45). c-Abl is a non-receptor-type tyrosine kinase that plays an important role in cell proliferation, migration, apoptosis, and fibrosis. c-Abl phosphorylates SKI-interacting protein (SKIP), a nuclear cofactor of the transcription factor Smad3. The c-Abl inhibitor imatinib suppressed TGF-β-induced expression of Smad3 targets as well as SKIP/Smad3 interaction (45). TGF-β-stimulation induced tyrosine phosphorylation of SKIP, and this phosphorylation was suppressed by imatinib. Tyr292, Tyr430, and Tyr433 residues in SKIP were shown to be involved in c-Abl-mediated phosphorylation. Phosphomimetic glutamic acid substitution at Tyr292 in SKIP enhanced, whereas its phospho-dead phenylalanine substitution attenuated TGF-β-induced SKIP/Smad3 interaction (45). Moreover, the phosphomimetic mutant of SKIP augmented transcriptional activity of Smad3. These results suggest that c-Abl phosphorylates SKIP mainly at Tyr292 and promotes SKIP/Smad3 interaction for the full activation of TGF-β/Smad3 signaling (45). What's more, SKIP can act on Core Binding Factor 1 (CBF1), thus inhibiting transcription in the Notch-mediated signaling pathway required for tumor growth (46). A recent work found that the FOXN3 regulated TGF-β/smad cell signaling pathway modulating the proliferation and differentiation of hematopoietic stem cells, and closely associated with the development of leukemic cells (47). However, it remains elusive whether FOXN3 mediates or causes the malignant transformation (through the TGF-β/smad cells signaling pathways).

## FOXN3 and Tumourigenesis

A meta-analysis of the FOXN3 gene (using the oncomine database) demonstrated that among 15 different malignancies (including liver cancer, lung cancer, colon cancer, prostate cancer, laryngeal cancer, glioblastoma multiform, and lymphoma) there was a very marked down-regulation of the FOXN3 gene. Taken together, this supports the hypothesis that the regulatory network including FON3 is indeed valid, even across diverse cancer types, and could serve as a guide for future strategies aimed at combating colorectal cancer metastasis. Also, this suggests that FOXN3 may be an important tumor suppressor gene (48, 49).

### FOXN3 in Breast Cancer

Nuclear paraspeckle assembly transcript 1 (NEAT1), located on chromosome 11q13.1, is a lncRNA that encodes two transcriptional variants, namely NEAT1-1 (3.7 kb) and NEAT1-2 (23 kb). Previous studies have revealed that NEAT1 functions as an essential structural component of a nuclear domain called paraspeckle, which participates in gene regulation mainly by nuclear retention of proteins and RNAs (50). Cumulatively, numerous studies have identified the aberrant expression and prognostic value of NEAT1 in various types of tumors including breast cancer (51), colorectal cancer (52), liver cancer, ovarian cancer (53), cervical cancer (54), gastric cancer (55), prostate cancer (56), lung cancer (57), and papillary thyroid cancer (58), the majority of which suggest NEAT1 as an oncogene that is overexpressed in tumors compared to their respective normal tissues and promotes tumor cell progression. This lncRNA is retained in the nucleus where it forms the core structural component of the paraspeckle sub-organelles. It may act as a transcriptional regulator for numerous genes, including some genes involved in cancer progression. Diseases associated with NEAT1 include Relapsing-Remitting Multiple Sclerosis and Dengue Disease. NEAT1 could mediate TGF-β1 expression by competitively sponging miR-339-5p (59). NEAT1 induced osteosarcoma cell proliferation and cell mobility by binding to miR-339-5p and increasing TGF-β1 in osteosarcoma (59). NEAT1 can function as a ceRNA by sponging hsa-mir-139-5p (60). The authors speculated that NEAT1 can modulate TGF-β1 expression by sponging hsa-mir-139-5p in hepatocellular carcinoma. These data indicates that targeting the NEAT1/hsa-mir-139-5p/TGF-β1 axis could be a new strategy some cancers (60).

Importantly, NEAT1 was found significantly up-regulated in breast cancer tissues and cell lines (61). NEAT1 can promote the growth of breast cancer cells by interacting with miR-101, which acts as tumor suppressor in several cancers by directly targeting EZH2 (61–63). Supporting this, Zhang et. al. found that NEAT1 was highly expressed in breast cancer tissue, and the NEAT1 expression correlated to tumor size and lymph node metastasis (64). This suggests that increased NEAT1 expression can act as an oncogene (in breast cancer), promoting proliferation and metastasis of breast cancer (64).

The scaffolding protein Switch-Independent 3A (SIN3A) has been implicated in breast cancer development (65, 66). SIN3A contains paired amphipathic helix (PAH) domains, which are important for protein-protein interactions and may mediate repression by the Mad-Max complex. Diseases associated with SIN3A include Witteveen-Kolk Syndrome and Rett Syndrome. Among its related pathways are C-MYB transcription factor network and SMAD Signaling Network. GO annotations related to this gene include DNA binding transcription factor activity and transcription factor binding.

Interestingly, Li et al. reported that NEAT1 was essential for FOXN3 interactions with the SIN3A complex. NEAT1 was found to be induced by estrogen in breast cancer cells (MCF-7), where. ERα (Estrogen receptor alpha) knock-down resulted in repression of NEAT1 induction. ChIP-Seq experiments found that the FOXN3-NEAT1-SIN3A complex suppressed a group of genes, including GATA3 and TJP1 (genes regulating cell maintenance and differentiation). It was found that ERα signaling and FOXN3-NEAT1-SIN3A complex function in an inhibitor feedback loop, under mammary epithelial cell growth ERα induced NEAT1 leading to SIN3A and FOXN3 mediated inhibition of GATA3 and TJP1 expression. Conversely, when conditions favored differentiation or the maintenance, ERα signaling reduced the growth signal. This regulatory mechanism contributed to the balance or choice (between differentiation or maintenance) of mammary epithelial cells. NEAT1 is induced by estrogen and participates in transcription regulation by the FOXN3-NEAT1-SIN3A complex, transcriptional targeting of ERα by the FOXN3-NEAT1-SIN3A complex implies that there exists a negative-feedback loop in ERα+ breast cancer cells between ERα and the FOXN3-NEAT1-SIN3A complex, in which ERα transactivates NEAT1, which is assembled into the FOXN3-NEAT1-SIN3A complex, and this complex, in turn, transrepresses ERα. The balance in this regulatory mechanism is required for normal development of mammary epithelial cells. The disturbance in this balance leads to breast cancer development and metastasis. The up-regulated FOXN3-NEAT1-SIN3A complex promotes EMT and invasion of breast cancer cells *in vitro* and the dissemination and metastasis of breast cancer *in vivo* (32).

### FOXN3 in Liver Cancer

Katoh et al. employed the Array-CGH technique to elucidate the characteristics of the overall chromosomal mutation of HCC and revealed the new hepatocellular carcinoma-associated genes, FOXN3 and caspase3. By using such methods as hierarchical clustering, according to HCC, they assigned 2 subgroups according to the chromosomal variation patterns, and investigated its relationship with the relevant clinical data, further confirm that HCC can be divided into genetically similar subgroups based on the heterogeneity of genetic spectra, and that the optimal therapeutic drugs for various HCCs can be expected to be developed based on the different types of genome chromosome of HCC (67). Sun et al. detected the expressions of FOXN3 in HCC and the matched normal tissues by means of real-time quantitative PCR and Western blot, and they found that FOXN3 was significantly down-regulated in HCC. In both *in vivo* and *in vitro* experiments, FOXN3 can inhibit the proliferation of liver cancer cells significantly (68). In addition, further mechanistic studies have shown that the forkhead box region of FOXN3 can bind to the promoter of the E2F5 transcription factor and inhibit the expression of E2F5 mRNA and protein, thereby inhibiting the proliferation of HCC cells. This mechanism is expected to be a therapeutic target for liver cancer (68).

### FOXN3 in Lung Cancer

The toll-like receptor (TLR) is one of the innate immunospecific receptors, and the TLR9 signals in both *in vivo* and *in vitro* experiments could promote the metastasis of human lung cancer cells (69). Given that CpG oligo-deoxynucleotides (CpG ODNs) can activate the TLR9 signaling pathways effectively in human lung cancer cells, they used the miRNA microarray technique to detect the expression of miRNA of 95D cells treated or untreated with CpG ODNs and look for the differentially expressed miRNAs after treatment with CpG ODNs. They found that the expression level of miR-574-5p was significantly up-regulated with the increase in CpG ODNs dose and the eclipse of act time. To clarify the potential effects of miR-574-5p on human lung cancer cells under the influence of TLR9 signals, researchers used the TLR9 signaling inhibitor chloroquine and the MyD88 inhibitory peptide to block the signaling pathway in both *in vivo* and *in vitro* experiments, and found that both chloroquine and MyD88 inhibitory peptide could significantly inhibit the up-regulation of CpG ODN-induced miR-574-5p in 95D cells, further confirming that miR-574-5p is regulated by the TLR9 signaling pathway. Further *in vitro* and *in vivo* experiments have shown that miR-574-5p can promote the proliferation of the human lung cancer tumor cells. In order to determine the exact mechanisms underlying miR-574-5p in the progression of TLR9 signaling-induced human lung cancer tumors, they selected multiple possible target genes (calcoco1, Rfx4, CD96, chex1, FOXi2, dgkg, znf589, zdhhc14, ccdc88c) using Target Scan and Miranda and conducted real-time quantitative PCR analysis. They found that the expression levels of FOXN3 mRNA and protein were significantly increased in the 95D cells transfected with the miR-574-5p inhibitor; they also found in the luciferase reporter assay that FOXN3 is a target gene of miR-574-5p. It can lead to the G0/G1 phase arrest by regulating the cell cycle of the human lung cancer cells, and affect the expression of CDK2 to further regulate the proliferation of the lung cancer cells, providing a new potential therapeutic target for the treatment of cancer (70).

### FOXN3 in Colon Cancer

Given Sun's previous research results (68), they found when using the oncomine database to analyze the sample datasets in the normal colon tissue and colon adenocarcinoma tissue that compared with the normal tissues, the expression of FOXN3 was decreased in colon adenocarcinoma tissue. By using such techniques as fluorescence quantitative PCR, Western blot and immunohistochemistry, they found that the mRNA and protein levels of FOXN3 were decreased in the colon cancer tissues. In the subsequently different pretreated colon cancer mouse model experiments, Western blot revealed that FOXN3 was expressed in the normal colon tissues and tumors, demonstrating the reduction in the level of FOXN3 protein. To further explore the effect of FOXN3 in the progression of colon cancer, they overexpressed and knocked down the FOXN3 gene in colon cancer cell line, and then used such experimental methods as MTT, soft agar assay, cell migration assay, cell invasion, and flow analysis, whereby they confirmed that FOXN3 could inhibit the proliferation, migration, and invasion of colon cancer cells and inhibit cell cycle progression. To explore the potential mechanisms underlying FOXN3 inhibition of the growth, migration and invasion of colon cancer cells, they used luciferase reporter gene assay to detect the activity of FOXN3 in various signaling pathways, whereby they found that the overexpression of FOXN3 inhibited the activity of Top flash (the target gene for the β-catenin/TCF signaling pathway when treating was performed using lithium chloride). In addition, they found by silencing FOXN3 that the expressions of N-cadherin, Snail, and c-Myc (three downstream genes of the β-catenin/TCF signaling pathway) were up-regulated and they promoted the activation of the β-catenin/TCF signaling pathway. This results further confirmed that the down-regulation of FOXN3 activates the β-catenin/TCF signaling pathway, thereby promoting the growth, migration and invasion of the colon cancer tumor cells. TCF4 binds to β-catenin to initiate transcription in the nucleus (33). Through the GST pull-down assay, they found that FOXN3 and β-catenin could form a complex in the colon cancer cell lines. By using the immunoprecipitation assay in cell lines overexpressing FOXN3 and β-catenin subsequently, they found that endogenous β-catenin and FOXN3 could also form a complex. At the same time, they used the co-immunoprecipitation assay in the normal cell line 293T, and discovered that the exogenously expressed FOXN3, TCF4, and β-catenin could form complexes. Then, they used immunoprecipitation in colon cancer cell lines, and found in the FOXN3 antibody-positive results that the capability of TCF4 to form complexes with β-catenin was weaker than in the FOXN3 antibody-negative results, showing that FOXN3 inhibits the catenin/TCF signaling pathway by blocking the binding between β-catenin and TCF4. To investigate the effect of FOXN3 in the regulation of metastasis of colon cancer cells, they silenced the FOXN3 gene and injected the luciferase-labeled colon cancer cells into nude mice by way of the left ventricle. Monitoring revealed that FOXN3 was down-regulated, which promoted the metastasis of the colon cancer cells, and immunohistochemistry showed that knocking out the expression of FOXN3 promotes tumorigenesis of colon cancer cells in the colonic tissues. This study showed that FOXN3 can inhibit the progression of the colon cancer, and that restoration of the function of FOXN3 is expected to become a novel therapeutic strategy for the colon cancer (71).

### FOXN3 in Laryngeal Cancer

In the laryngeal cancer and paracancerous mucosa specimens, gene chip data analysis showed that the FOXN3 gene was significantly down-regulated while the ADAM12 gene expression was significantly up-regulated, and subsequent real-time quantitative PCR for the validation of the mRNA expressions in 24 cases of laryngeal cancer and 17 cases of normal mucosa showed that the results were consistent with the those of detection with gene chip. As a result of the high expression of ADAM12 and the low expression of CHES1 in laryngeal carcinoma, they analyzed the presumed diagnostic values of the ratios of expressions of CHES1 and ADAM12 genes by setting the threshold of the ratio of the two genes (ADAM12/CHES1) at 0.4. After validation in 24 laryngeal cancer and 17 normal mucous membranes, they found that the ratio of the two genes could be used to distinguish between tumor and normal tissues, with the correct validation rate being as high as 70%. Therefore, it is expected that the ratio of ADAM12/CHES1 can be taken as a molecular marker to distinguish between tumor and non-tumors, and that it may become the best diagnostic indicator for laryngeal cancer (72).

### FOXN3 in Hematological Malignancies

The abnormal expression of FOXN3 protein is of great significance in hematological tumors. When Stefan Nagel et al. used the gene chip technique to find abnormal expressions of the FOX gene in the Hodgkin lymphoma (HL) cell lines and patients, they found that the expression levels of the FOXC1 gene and the FOXD gene were increased, and that the transcriptional levels of FOXN3, FOXO1, and FOXP1 genes were reduced. They discovered when using enrichment analysis to explore the mechanisms underlying the regulation of FOX gene expression that there was a potential activation of the JAK-STAT signaling pathway when FOXD1 and FOXN3 regulate transcription. In further studies, they found that TGF-β could inhibit the expression of FOXD1 in HL cell lines pretreated with IL13, TGF-β, and WNT5B using RQ-PCR, and that the WNT signaling pathway could reduce the expression of FOXN3. Therefore, they determined that FOXD1 and FOXN3 are affected by specific signaling pathways, and that these pathways have already shown abnormal activity in HL (73).

FOXN3 protein can inhibit the expression of the PIM2 gene and protein biosynthesis, thereby regulating cell proliferation (73); similarly, studies on three different types of lymphomas revealed that the expression of CHES1 in primary exudative lymphoma, diffuse large B-cell lymphoma and hairy cell leukemia was reduced. After overexpression of FOXN3 in lymphoma cell lines, they found through qPCR, Western blot and immunoprecipitation found that CHES1 can bind to the PIM2 gene directly to reduce the phosphorylation level of 4EBP1, the target of PIM2, leading to a decreased level of PIM2 and thus promoting tumorigenesis. This study confirmed that in lymphomas, the deletion of FOXN3 leads to increased level of the target gene PIM2, thereby weakening the inhibitory effect of FOXN3 on tumor growth. Specifically, PIM2 represents an important oncogene as well as the therapeutic target for multiple solid tumors and hematological tumors (74, 75).

In a study on the changes in the expression levels of genes of T-cell large granular lymphocyte leukemia (T-LGL), they used such research methods as multicolor fluorescence *in situ* hybridization, gene sequencing and LM-PCR and found that there was an increase in FOXN3 gene expression in a T-LGL patient with gene amplification in 14q, indicating that sub-genomic rearrangement alters the gene expression in patients with T-LGL, and that it is associated with the pathogenesis and survival pattern of T-LGL (76).

In a related study on the T-cell leukemia tumor suppressor network, the FOXN3 gene was down-regulated in T-lymphocytic leukemia (T-ALL) significantly. Analysis of the target genes revealed that the homologous gene ZHX1 could be directly activated by FOXN3. In T-ALL, a decreased expression of FOXN3 can lead to decrease in the level of ZHX1, while ZHX1 is mainly expressed in the normal T cells. Its homologous gene ZHX2 is more vigorously expressed in the normal B cells, and ZHX1 and ZHX2 represent the TS gene in both T and B cell malignancies, respectively. Generally speaking, the TS gene regulates proliferation and apoptosis of cells via synergy with other TS genes or oncogenes, indicating that FOXN3 and ZHX1 form a regulatory network in the development of T cells and the genesis of leukemia. This also shows that FOXN3 can interact with other transcription factors or transcriptional regulators to inhibit transcription indirectly, jointly regulating proliferation and differentiation of cells (77).

Zhang et al. used the high-resolution aCGH technique (resolution, 2kb) to scan the bone marrow leukemia cell genome in 24 patients with acute myeloid leukemia-M5(AML-M5), and found 2 (8.3%) patients had deletion of chromosome 14 involving the FOXN3 gene. Then, they employed qRT-PCR to detect the expression levels of FOXN3 in the bone marrow of 97 cases of acute leukemia [78 AML cases, and 19 acute lymphocytic leukemia (ALL) 19 cases] and 16 normal humans, and found that the expression levels of FOXN3 in patients with ALL and AML were decreased to varying degrees, especially in AML patients in whom the expression level of FOXN3 was significantly different from that in the normal controls. Thus, it can be concluded that FOXN3 may serve as an anti-oncogene in AML (78).

## Conclusion

FOXN3 is mostly down-regulated in most solid tumors and hematological malignancies, and up-regulated in only a small number of diseases. However, the specific functions of FOXN3 in the cells of mammals, including humans, and its mechanisms of action in tumor cells have not yet been clearly confirmed. In particular, the mechanisms of action of FOXN3 in AML is still elusive, and whether FOXN3 mediates the malignancy transformations of hematopoietic stem cells through the TGF-beta/smad cell signaling pathway is still unknown. We now speculate that FOXN3, as a tumor suppressor gene, is involved in the development of leukemia. The deletion or mutation of the FOXN3 gene may lead to a decreased level of FOXN3 expression and affect the formation of the FOXN3-SKIP complex, thereby resulting in the relatively increased inhibition of the smad2/3 transcription complex by Ski. Inhibition of the negative regulation of the TGF-beta-mediated signaling pathway on cell proliferation leads to imbalanced proliferation and differentiation of the hematopoietic stem cells and induces leukemia. In the future, it is expected to further elucidate the molecular biology and cytogenetic basis of a low expression of FOXN3 in patients with AML. In addition, the mechanisms of the targeted protein of the FOXN3 transcription factor may be discovered through further studies, which will give insight into the novel tumor suppressor gene for the complex network of the development of leukemia.

## Author Contributions

XK, JZ, CY, YS, JW, XB, JB, and YF wrote and edited the paper. XK, JB, and YF final approval of the paper.

### Conflict of Interest Statement

The authors declare that the research was conducted in the absence of any commercial or financial relationships that could be construed as a potential conflict of interest.

## Abbreviations

FOXN3: forkhead box N3
CHES1: check point suppressor 1
FOX: forkhead box
HTLF: human T-cell leukemia factor
ILTR: long-chain terminal repeat
PDGFA: platelet-derived growth factor A
SFTPB: surfactant-associated protein B
MCC: multi-center cell
GO: Gene Ontology
HCC: hepatocellular carcinoma
mRNA: messenger RNA
TGF-β: transforming growth factor β
BMP: bone morphogenetic protein
SBE: Smad binding element
SKIP: Ski-interacting protein
CBF1: Core Binding Factor 1
NEAT1: nuclear paraspeckle assembly transcript 1
SIN3A: Switch-Independent 3A
PAH: paired amphipathic helix
ERα: Estrogen receptor alpha
TLR: toll-like receptor
CpG ODNs: CpG oligodeoxynucleotides
HL: Hodgkin lymphoma
T-LGL: T-cell large granular lymphocyte leukemia
AML: acute myeloid leukemia
ALL: acute lymphocytic leukemia.
